# Frailty in Older Adults with Dengue Fever

**DOI:** 10.3390/medicina60040537

**Published:** 2024-03-26

**Authors:** Yu-Sheng Hu, Yu-Tai Lo, Yi-Ching Yang, Jiun-Ling Wang

**Affiliations:** 1 Department of Geriatrics and Gerontology, National Cheng Kung University Hospital, College of Medicine, National Cheng Kung University, Tainan 701, Taiwan or wither0522@gmail.com (Y.-S.H.); n105218@mail.hosp.ncku.edu.tw (Y.-T.L.); 2 Department of Internal Medicine, National Cheng Kung University Hospital, Tainan 701, Taiwan; 3 Department of Medicine, College of Medicine, National Cheng Kung University, Tainan 701, Taiwan

**Keywords:** dengue, elderly, functional decline, frailty, acute geriatric unit care

## Abstract

*Background and objectives:* Dengue is one of the most common epidemic infections around the world. Dengue infections in older adults are related to an atypical presentation and a high mortality. Frailty is associated with poor recovery from hospitalization due to infection. However, few studies describe frailty and functional decline after dengue infection. The current case series study aims to investigate the baseline frailty status, functional decline, and time to recovery in older adults after dengue infection. *Method:* We studied seven patients with post-dengue frailty who had been admitted to the geriatric ward in one tertiary medical center in Taiwan during the 2023 dengue fever outbreak. *Result:* The mean age was 82 years old. The clinical frailty scale worsened from a mean of 4.7 at baseline to 6.3 at dengue diagnosis. The mean Katz Index of independence in activities of daily living decreased from 10.6 at baseline to 4.7 with dengue, and it recovered to 6.7 one month after discharge. *Conclusions:* Our preliminary data suggest that there is indeed an increase in frailty in older adults due to dengue. Post-dengue frailty and functional decline might be profound and persistent. Acute geriatric care intervention rehabilitation for frailty after dengue may benefit this population.

## 1. Introduction

Dengue is one of the most common epidemic infections around the world, estimated at 50 million infections per year [1,2]. Most infections are subclinical [3]. For some patients, dengue may manifest as nausea, vomiting, fatigue, anorexia, abdominal pain, weakness, and even life-threatening organ failure [1,2]. In recent dengue epidemiology, higher death rates were observed in the <5-year-old and >70-year-old age groups. However, mortality decreased gradually among those <5 years old and increased gradually among those >70 years old [4]. In Taiwan, the geriatric population (aged ≥65 years) will be more than 20% by 2025 [5]. The shift in dengue to adults and older adults has raised awareness in Taiwan. And older adults have experienced the highest incidences of dengue fever, dengue hemorrhagic fever, and dengue virus-related deaths [5,6]. Older dengue patients also present atypically and are at higher risk of an excess of hospitalization [7].

Global population aging is rapidly accelerating, and frailty poses a significant challenge associated with aging. Frailty serves as a predictor of mortality and post-discharge functional status [8]. It is characterized by increased functional dependency and slow recovery post-new illness or minor stress, suggesting inadequate functional reserves [8]. The experience of functional decline and catastrophic disability and its association with frailty has been reported for influenza and other acute respiratory illnesses [9]. After the 2019 COVID epidemic, many studies showed older adults had worse outcomes and increased rates of functional decline [10]. Patients suffer from chronic symptoms after COVID-19 infection, which can become debilitating and affect the daily routine [11]. And frailty was associated with an increased risk of developing post-acute sequelae of COVID infection [12].

More than half of the elderly patients who recovered from COVID-19 experienced functional decline upon discharge [13]. Given that both dengue fever and COVID-19 are viral diseases correlated with inflammation, we hypothesize that dengue fever may similarly result in functional decline. In contrast to SARS-CoV-2 and influenza infection, most of the literature discussing older adults with dengue only focuses on high mortality [14,15]. There were reports from dengue patients that the proportions of patients who reported persistent symptoms were likely to experience work loss from 1 week to 2 years after hospital discharge. Persistent symptoms typically occur in females of all ages or in older adults [16]. After 3 months of infection, among the dengue fever population, fatigue, attention deficits, and memory impairment are the most common chronic symptoms, and the burden caused by chronic sequelae following dengue is often underestimated. However, it is currently unclear what the driving factors of these chronic sequelae are [17]. Fatigue following infection has also been observed in cases of Lyme disease and Epstein–Barr virus infection, but the underlying pathogenesis remains unclear [17].

From the literature review, none of the literature reported the frailty status or functional decline after dengue infection. A study from Belize reported weakness and loss of appetite as the main manifestation of dengue in the geriatric population [18]. And another study in Taiwan showed a higher risk of dementia after dengue infection [19]. However, there is no relevant article describing in detail the frailty status and functional decline in geriatric patients during the timeline of dengue infection. Very few studies to date have addressed the effects of dengue on the onset of, progression of, and recovery from frailty. Here, we report a small case series of dengue infection with frailty status and functional decline during the dengue outbreak in Taiwan in 2023. Due to the lack of consensus regarding the significance of frailty in young and middle-aged adults, we focused on dengue fever in the elderly group.

## 2. Materials and Methods

### 2.1. Diagnosis and Management of Dengue Fever

There were 26,703 confirmed dengue infections in 2023 in Taiwan. In Taiwan, all suspected dengue cases must be reported to the Taiwan Centers for Disease Control (CDC), and their specimens must also be sent to the Taiwan CDC for confirmation through the official infectious disease surveillance system. The confirmation diagnosis in Taiwan involves examination of the serology markers (IgM and IgG), real-time polymerase chain reaction (RT-PCR), and NS1 antigen [20]. In Taiwan, the criteria for hospitalization due to dengue infection include the presence of positive danger signs, such as persistent vomiting, severe abdominal pain, rapid breathing, bleeding, cold, sweaty skin or cold hands and feet, and changes in consciousness. Additionally, laboratory findings of increasing hematocrit concurrent with a rapid decrease in platelet count require immediate hospital admission.

### 2.2. Geriatric Ward Characteristics and Admission Criteria

National Cheng Kung University Hospital is a 1135-bed tertiary care medical center in southern Taiwan. The geriatric ward at National Cheng Kung University Hospital integrates a multidisciplinary team comprising geriatric physicians, nurses, social workers, occupational therapists, physiotherapists, pharmacists, dietitians, care managers, and others. This team conducts weekly multidimensional case discussions upon the domains of comprehensive geriatric assessment (CGA) to address treatment, medication, nutrition, rehabilitation, care issues, and family burden for inpatients, followed by the provision of appropriate assistance. The geriatric ward design features an accessible environment, including essential accessibility features and proactive measures to minimize risks, hazards, and actively foster a senior-friendly environment.

Conditions suitable for hospitalization in the geriatric ward in our institutions include the following: 1. individuals requiring CGA due to complex and multiple healthcare issues or comorbidities, including psychological and social issues, necessitating complete inpatient evaluation; 2. those with mild acute illnesses combined with geriatric syndromes (e.g., delirium, dementia, depression, incontinence, malnutrition, mobility impairment, falls, pressure ulcers, insomnia, dizziness, and polypharmacy); 3. older patients who have the potential to recover their activities of daily living through active geriatric assessment, management, treatment, and rehabilitation during hospitalization. 4. individuals aged 65 and above, mostly over 75, with mild acute illnesses. Conditions unsuitable for hospitalization in the geriatric ward include the following: 1. those unable to regain function despite active and CGA and management; 2. individuals requiring special care by a specialist due to single-organ system issues; 3. patients with severe conditions requiring intensive care unit admission; 4. those requiring palliative care due to end-stage diseases; 5. patients awaiting transfer to long-term care facilities.

### 2.3. Participants

We retrospectively collected the cases from 1 September to 31 November 2023 of patients who were admitted to the geriatric ward for post-dengue frailty during the 2023 dengue outbreak in one tertiary medical center in southern Taiwan. Following fluid therapy during the critical phase in the emergency room and internal medicine ward, patients in the convalescent phase may be transferred to the geriatric ward if frailty is identified. In our protocol, the geriatric department may receive a consultation from the internal medicine department when asked for evaluation of geriatric syndrome including frailty and functional decline. The criteria of geriatric ward transferal had been described as suitable for hospitalization in the geriatric ward above. Patients who were less than 65 years old or had ICU admission or were receiving mechanical ventilation were excluded.

### 2.4. Studied Variables

The primary outcome was activities of daily living (ADLs) at baseline, at the initial stage in the geriatric ward, and 1 month post-discharge follow-up ADLs. Performance of ADLs was measured at different time points according to the Katz ADL scale [21]. The Katz ADL is an ordinal scale used to measure performance and level of dependence in daily living. The scale includes disabilities causing an individual’s ability to perform essential self-care tasks. Six tasks representing ADL and mobility are scored (eating, bathing, dressing, toileting, transferring, and continence) with 0 (dependence), 1 (partial dependence), and 2 (independence). A higher number is a reflection of greater ability to function independently in daily living.

Data on demographic characteristics, namely age and sex, were collected upon the inclusion of participants. Age was a continuous variable; sex was a dichotomous variable with male and female. Clinical data, including past medical history, previous hospitalization, emergency room (ER) visit in the past 6 months, initial presentation of dengue fever, and diagnostic methods were collected. Frailty status was also collected through chart review.

The change in the frailty status from baseline (before dengue) and at the geriatric ward was also reported using the clinical frailty scale (CFS) [22]. During hospitalization, the trained healthcare workers (including geriatric physicians and nurses) interviewed patients and their family members, asking them to recall the activities of daily living prior to the onset of illness, occurring 1 month ago, as a reference baseline point. The CFS includes a 9-point scale to summarize the overall level of frailty of an older adult after they have been evaluated by a healthcare professional, and higher scores mean greater risk of frailty. The CFS involves a graded assessment by clinicians, based on a comprehensive evaluation of the older individual’s functional status and disease severity, with nine grades ranging from 1 (very fit) to 9 (terminally ill). Patients scoring ≥5 were categorized as frail. In patients with CFS of 5, they have limited dependence on others for instrumental activities of daily living [23]. In our study, experienced healthcare workers in the geriatric ward routinely evaluated patients’ frailty status using the CFS.

The process of geriatric care requires individualization. We considered that understanding every patient’s functional and frailty status would help the multidisciplinary team to plan personalized medication regimens. Therefore, we collected baseline data from the CGA during admission, conducted by our nurse practitioners and case managers.

These nursing staff undergo professional training in geriatrics before carrying out this assessment.

CGA is defined as a multidimensional interdisciplinary diagnostic process to search for beneficial intervention to improve the outcome in the geriatric population [24]. The focus is on identifying the medical, psychological, social, and functional capability of frail older adults. The domains of CGA include frailty, delirium, cognition, depression, nutrition, functional status, falls, polypharmacy, healthcare utilization, physical function, and care issues [24]. We evaluated cognitive function, according to the Short Portable Mental Status Questionnaire (SPMSQ) [25], which is a brief cognitive tool designed to screen cognitive impairment in geriatric inpatients and outpatients. SPMSQ contains 10 questions, and we dichotomized the cognitive function into normal and abnormal (having more than 3 errors in 10 questions). Nutritional status was measured according to the Mini Nutritional Assessment—Short Form (MNA-SF), which is a screening tool with a maximum of 14 points to help identify older patients who are malnourished or at risk of malnutrition [26]. Delirium status was measured according to the Confusion Assessment Method (CAM), which is a standardized evidence-based tool that enables non-psychiatrically trained clinicians to identify and recognize delirium quickly and accurately in both clinical and research settings. The CAM includes four features found to have the greatest ability to distinguish delirium from other types of cognitive impairment [27].

Mobility was measured according to the Timed Up and Go test (TUG) and Short Physical Performance Battery (SPPB) [28,29]. The TUG test measures how long it takes to stand up, walk a distance of 3 m, turn, walk back, and sit down again [28]. Mobility is assessed based on time to complete the test (normal: <10 s; good mobility: 10–20 s; walking and balance problems: 20–30 s). The SPPB is made up of a set of three tests: standing static balance in three positions; lower limb strength and power through getting up and sitting on a chair; and walking speed at normal pace. Each test is scored from 0 (inability to perform the task) to 4 points (best performance). The SPPB total score ranges from 0 (worst performance) to 12 points (best performance) and categorically evaluates performance in the tests using three or four classes of scores: three classes: 0–6 points (poor performance), 7–9 points (moderate performance), and 10–12 points (good performance); or four classes: 0–3 points (disability/very poor performance), 4–6 points (poor performance), 7–9 points (moderate performance), and 10–12 points (good performance). Lower SPPB scores (≤9) were associated with an increased risk of death compared to higher values (scores of 10–12).

### 2.5. Statistical Analysis

To analyze the data related to changes in CFS and ADL scores, we employed the Student’s *t*-test within the cohort of dengue fever patients using Excel. This statistical analysis ensured a comprehensive evaluation of observed differences. The patient outcomes, following meticulous follow-up, offered detailed insights into variations in both CFS and ADL. The resulting data reflect a methodologically sound approach that enhances the reliability and validity of the insights into the impact of dengue fever on frailty and daily functioning. A *p*-value less than 0.05 indicates statistical significance.

### 2.6. Ethical Approval

The ethics committee of National Cheng Kung University approved this study (approval number: B-ER-111-214). Patient consent was waived due to the retrospective nature of the study, and the analysis used anonymous clinical data.

## 3. Results

**Figure 1:**
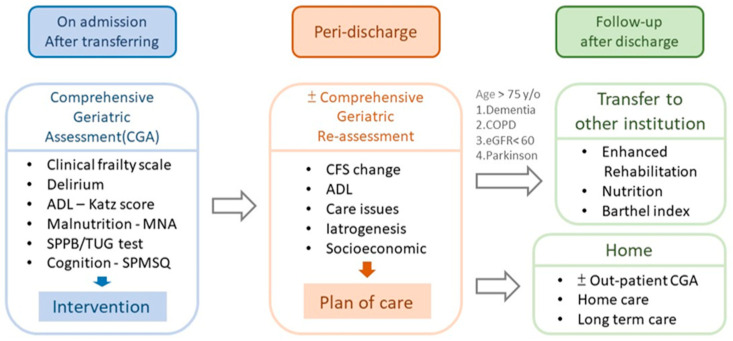
Multidisciplinary care and follow-up in geriatric care. Note: ADLs, activities of daily living; COPD, chronic obstructive pulmonary disease; eGFR, estimated glomerular filtration rate; CFS, clinical frailty scale; MNA, mini nutrition assessment; SPPB, Short Physical Performance Battery; TUG test, Timed Up and Go test; SPMSQ, Short Portable Mental Status Questionnaire.

The process for patients in our acute geriatric unit care is shown in Figure 1.

We collected seven patients’ data retrospectively, and most of the patients were transferred to the geriatric ward. The demographic data of these seven patients are shown in Table 1.

The mean age was 82 years old. Baseline mean activities of daily living (ADLs), according to the Katz score (taking meals, dressing, transition, toileting, continence, taking a bath; total independence as 2, partial dependence as 1, total dependence as 0 in each domain), were 10.6, and the post-dengue mean ADL was deteriorated to 4.7 (*p* = 0.001). All the patients had a complete geriatric assessment (CGA), enhanced nutrition support, and rehabilitation as the protocol in our geriatric ward. Details of the CGA domain score are shown in Table 2.

**Figure 2:**
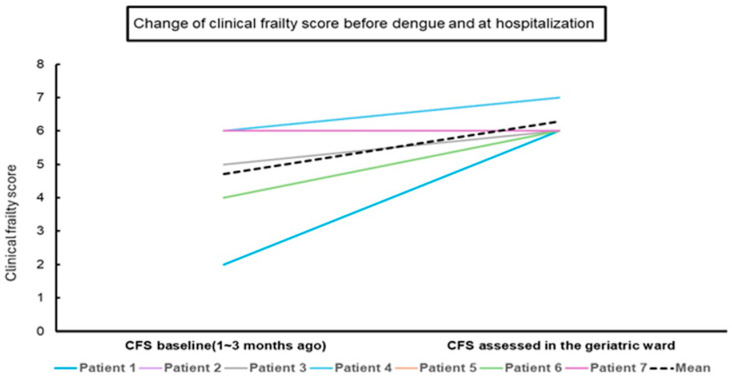
Change in the clinical frailty score from before dengue to hospitalization.

Figure 2 shows the CFS change from the baseline score before dengue to evaluation in the geriatric ward during hospitalization. The baseline CFS score demonstrated a mean of 4.7 ± 2.2, which increased to 6.3 ± 0.2 (*p* = 0.017) upon admission to the geriatric ward.

Post-dengue frailty and functional decline might be profound and persistent. The long-term outcome might be poor despite aggressive geriatric and post-acute care. One of seven patients died, and another two did not improve after intervention. Six of seven patients did not recover to their baseline ADL. Recovery or not by 1 month was associated with long-term outcomes [7]. Two patients were referred to other post-acute care (PAC) institutions due to frailty with functional decline.

**Figure 3:**
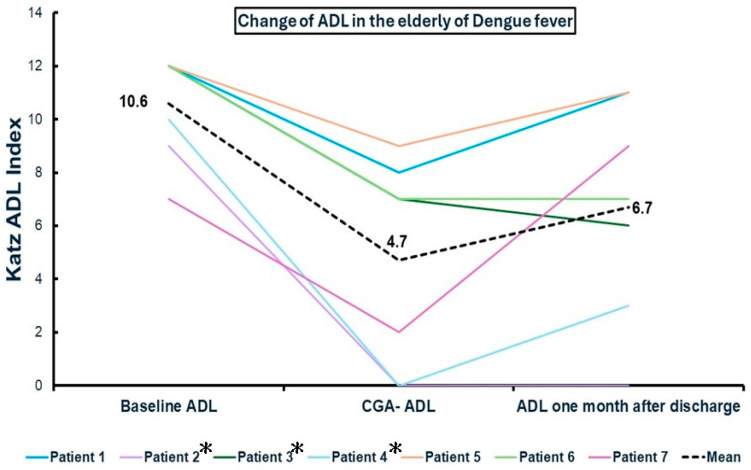
Change in the Katz ADL index from baseline before dengue, at hospitalization, and 1 month after discharge. Note: Patient 2 died from choking before the final assessment. CGA, comprehensive geriatric assessment; ADLs, activities of daily living. Patients 2, 3, 4 *: patients with poor Short Portable Mental Status Questionnaire score.

The one-month follow-up by phone call or records from the PAC unit reported that the mean ADL was 6.7 (one missing dataset because the patient died). Three of them had an improved ADL, two of them were stationary, and the other two patients had a worsened ADL one month later. Among these patients, one 85-year-old patient died 3 days after completing her PAC course, due to vomiting and asphyxia. One 91-year-old patient referred to another PAC program recovered well, and his Barthel index improved from 35 to 75. The complete data about the change in the ADL are shown in Figure 3.

In patients with poor Short Portable Mental Status Questionnaire score (patients 2, 3, 4), the change in ADL was 9–0–0, 12–7–6, and 10–0–3 (mean 10.3–2.3–3) (Figure 3, marked with asterisk *). We found the functional decline recovery to be poor, especially in patients with cognitive impairment.

The mean ± standard deviation in the Katz ADL index decreased from 10.6 ± 4.0 at baseline before dengue to 4.7 ± 15.2 at hospitalization (*p*-value = 0.001) (Figure 3). This indicates that there is indeed a statistically significant decline in the functional status of a group of elderly individuals following hospitalization for dengue fever. And the mean ± standard deviation in the Katz ADL index increased from 4.7 ± 15.2 at hospitalization during dengue to 6.7 ± 16.9 (*p*-value = 0.098 in the T-test) 1 month after discharge (Figure 3). The increase in the ADL index may not be statistically significant.

## 4. Discussion

We have documented cases of post-dengue frailty in older adults during the 2023 outbreak in Taiwan. The study encompasses a multitude of frailty indicators that reflect post-dengue dependency among elders. The detailed progression and the subsequent changes in functional decline after dengue fever were recorded. Our results demonstrate a notable decrease in the Katz ADL index from mean 10.6 at baseline before dengue to 4.7 at the time of hospitalization. Additionally, the index showed an increase from mean 4.7 at hospitalization during dengue to 6.7 one month after discharge. These observations highlight the dynamic changes in functional independence and daily living activities in dengue patients, reflecting the impact of the disease on patient outcomes over time. Senior-unfriendly structures and inefficient care could increase the risk of missed nursing care in hospitalized frail older adults in medical wards [30]. Our findings suggest that multidisciplinary geriatric care may aid in managing functional decline in elderly dengue patients.

Frailty is a multisystem aging syndrome characterized by a decline in physiological and functional reserves, affecting various tissues and organs due to aging-related physiological changes [31]. Traditionally, frailty is described as an age-related decrease in muscle strength and physiological function, rendering individuals vulnerable to dependence, vulnerability, and increased mortality risk [8]. A high incidence of functional decline after hospitalization for acute illness was observed, even worsening after discharge [9]. Previous study showed that relevant predictors of functional decline included age, previous hospitalization, depression, length of hospital stay, cognitive deficit, restraint, social support, not having a partner, and delirium [32,33]. However, from systemic review, there was no specific diagnosis found as a predictor of functional decline in the hospitalization of the elderly [34]. One acute illness commonly associated with functional decline in the elderly is acute respiratory infection (ARI), particularly pneumonia [35,36]. However, this concept was not reported specifically for patients with dengue fever. In our small cohort, most patients had comorbidities. Previous studies have indicated that comorbidities may result in increased dependence in activities of daily living (ADLs) following hospitalization for acute infections [37,38]. Due to the limited number of cases, it is difficult to ascertain which comorbid factors are associated with the severity of post-dengue frailty or functional decline.

Optimizing prevention of frailty and rehabilitation for functional decline is essential for all hospitalized older adults, including those with dengue fever [39]. Intervention for frailty included CGA, exercise, balance training, and nutrition [9,39,40]. CGA and multidisciplinary interventions have the potential to enhance health outcomes for older individuals who are at risk of experiencing declining health and hospital admissions [41]. In our geriatric ward, malnutrition Universal Screening Tool (MUST) was used as a routine screening protocol, and a dietician is consulted if the MUST score is more than 3. We also evaluated nutritional state with MNA-SF as part of CGA. If malnutrition risk is identified, the patient will initially be provided with oral nutritional supplements, followed by consultation with a dietitian. The previous literature has not investigated the nutritional assessment and prognosis of elderly patients with dengue fever.

A more recent study showed that there were slightly increased risks of non-vascular and overall dementia in dengue patients [19]. We did not know whether dementia was associated with post-dengue frailty. A number of epidemiological studies have reported that frailty increases the risk of future cognitive decline [42]. The reports of neurological sequelae of dengue fever are increasing [43]. The dengue virus can trigger an inflammatory cascade, resulting in endothelial dysfunction and subsequent plasma leakage [44]. This vascular dysfunction can potentially contribute to neurologic conditions such as stroke or dementia [45]. However, there is still no clear expert consensus on the classification of encephalopathy versus encephalitis syndromes in dengue infection. In the future, more research is needed to understand the prevalence of frailty among patients with dengue fever and its risk factors. It is also necessary to clarify the association between post-dengue frailty and the subsequent development of dementia. Identifying the most suitable intervention methods to prevent functional decline in older individuals after dengue fever is critical. From a different point of view, cognitive impairment also leads to poor recovery of disability after acute illness in our series. In one study, Gill et al. concluded that the probability of patients recovering to pre-hospital function is low for those who have been admitted to a nursing home and have experienced disability following acute hospitalization. Cognitive impairment is one of the negative confounders for recovery [46]. Cognitive impairment may be associated with functional decline in hospitalized older adults [47] and may increase length of stay, institutionalization rates, and even mortality [48].

Recent studies have also indicated an association between dengue fever and the future occurrence of stroke and cardiovascular disease [49,50]. Pre-frailty and frailty are independently associated with a higher risk of developing major adverse cardiovascular outcomes [51]. More study on the impact of a combination of therapies, including physical resistance exercises and pharmacological, nutritional, cognitive, and psychosocial interventions for preventing or treating frailty in older dengue patients is necessary. And we hope this early intervention can further decrease the risk of cardiovascular disease after dengue infection.

Frailty and pre-frailty are associated with higher inflammatory parameters and in particular CRP, TNFα, and IL-6 [52]. Furthermore, a pilot study involving thirteen pairs of age-, race-, and sex-matched frail and non-frail older adults living in the community, with a mean age of 84 years, revealed that frail participants exhibited increased counts of T cells expressing chemokine CC receptor-5 (CCR5) compared to the matched non-frail controls [53]. During dengue virus infection, there is an increased expression of CCR5 on CD4. Notably, an increased presence of T-cytotoxic type-1 cell-related CCR5+ cells among CD8 T cells was observed in the most severe dengue patients, distinguishing them from those without warning signs [54]. Due to the association between cytokines and the pathogenesis of dengue fever revealed in previous studies, as well as the correlation between changes in cytokines and frailty in older adults, future research to identify biomarkers, e.g., CRP or CCR5, that can predict frailty in elderly dengue fever patients is of great importance. As frailty is associated with chronic inflammation, further research is needed to understand whether immune-modulating medications, including steroids, or cholesterol-lowering statin medications [55], may serve as protective factors in reducing the occurrence of frailty in dengue fever.

The limitation of this study is the small number (*n* = 7) of cases/patients, and we lack a control group. We had no routine evaluation of the function and frailty status in every patient who was admitted or followed. And recall bias about health and function before the current illness of dengue fever may be possible [56].

The incidence of post-dengue frailty and functional decline was undetermined because not all patients with dengue and frailty were collected in our study. The acute care model for older dengue fever patients and its potential improvement for debilitated patients may need further research before being extrapolated from the limited patient experiences to a broader population in low-income tropical countries. We do not know whether the occurrence of frailty in these older individuals is related to commonly used medications, including sedatives that may impair central nervous system function. As there are no specific antiviral medications for dengue fever, there are few medications that may impact frailty during the acute phase, and at most, antihistamines may be prescribed by physicians due to itching.

While our case enrollment is limited, it is hoped that such research can serve as a springboard for prospective longitudinal studies to better understand the prevalence of frailty and functional decline in older individuals resulting from the chronic effects of dengue fever. Additionally, the factors associated with the occurrence of frailty, such as viral subtypes, viral load in the blood, cytokines in the blood, or pre-existing chronic diseases, need to be explored in correlation with the acute phase. This may open avenues for further interventions to address the occurrence of frailty.

## 5. Conclusions

This is the first case series of post-dengue frailty in older adults, and we aim to raise the necessity of the detection, grading, and management of post-dengue frailty and functional decline. Our series reported on the severity of frailty in older adults and the challenges for older adults with dengue fever. Nutrition, rehabilitation, and post-acute geriatric care may contribute to their recovery from frailty. However, one-month follow-up assessments of activities of daily living (ADLs) showed that six out of seven patients did not return to their baseline ADL levels, despite receiving optimal geriatric care in our hospital, especially among the patients with cognitive impairment. This result underscores the unmet need for screening and treating patients with post-dengue frailty and functional decline.

## Figures and Tables

**Table 1:** Demographic data of the seven patients. Note: CCI: Charlson Comorbidity Index, CFS: clinical frailty scale.

No.	Age	Sex	CCI	Past Medical History	Previous Hospitalization	Initial Presentation	Diagnostic Methods	CFS Baseline	CFS at Ward	Outcome
1	78	M	3	Nil	0	Fever and chills	NS1Ag +	2	6	Living independently
2	85	F	6	Hypertension, diabetes mellitus, previous stroke	0	Fever, chills, weakness, dyspnea	IgM+ IgG+	6	7	Death
3	82	M	5	Parkinsonism, history of subdural hemorrhage without residual sequelae	0	Fever and chills, altered mental status	IgM+ IgG+	5	6	Partial dependency
4	75	M	5	Hypertension, chronic kidney disease stage III, Alzheimer disease with Parkinsonism	1	Fever and weakness	NS1Ag +	6	7	Partial dependency
5	83	M	5	Diabetes mellitus, chronic kidney disease, stage III	1	Fever, chills, and weakness	NS1Ag +	4	6	Living independently
6	81	F	5	Diabetes mellitus, gout, L2 vertebral fracture status post-kyphoplasty	0	Weakness, hyperglycemia	NS1Ag +	4	6	Partial dependency
7	91	M	6	Diabetes mellitus, chronic kidney disease, stage IIIA, history of subdural hemorrhage, previous stroke	1	Fever and weakness	NS1Ag + IgM+ IgG+	6	6	Partial dependency

**Table 2:** Some CGA domain scores assessed in the geriatric ward. CGA: comprehensive geriatric assessment, CAM: Confusion Assessment Method, SPMSQ: Short Portable Mental Status Questionnaire, MNA-SF: Mini Nutritional Assessment—Short Form, SPPB: Short Physical Performance Battery, TUG test: Timed Up and Go test, NA: no data available.

No.	Baseline Katz ADLs	CGA- CAM-4	CGA- Katz ADLs	CGA- SPMSQ	CGA- MNA-SF	CGA- SPPB
1	12	0	8	10	12	7
2	9	3	0	0	1	0
3	12	1	7	2	8	4
4	10	1	0	2	7	0
5	12	0	9	9	10	NA
6	12	0	7	9	8	NA (TUG test = 52 s)
7	7	1	2	7	3	0
	0–12, refer to text	>2 = abnormal	0–12, refer to text	<8 = abnormal	12–14 = normal 8–11 = risk of malnutrition 0–7 = malnutrition	0–12; composed of balance test, gait speed test, and chair stand test

## Data Availability

Please contact the author for data requests.
